# QuickStats

**Published:** 2014-05-02

**Authors:** FROM THE NATIONAL CENTER FOR HEALTH STATISTICS

**Figure:**
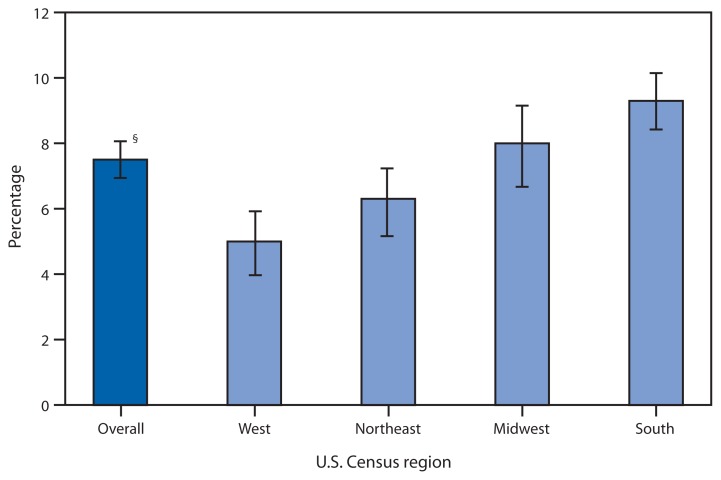
Percentage of Children Aged 6–17 Years Prescribed Medication During the Preceding 6 Months for Emotional or Behavioral Difficulties,* by Census Region — National Health Interview Survey,^†^ United States, 2011–2012 * Based on responses to the question, “During the past 6 months, was [child’s name] prescribed medication or taking medication for difficulties with emotions, concentration, behavior, or being able to get along with others.” ^†^ Estimates are based on household interviews of a sample of the noninstitutionalized, civilian U.S. population and are derived from the National Health Interview Survey sample child component. ^§^ 95% confidence interval.

During 2011–2012, among children aged 6–17 years, 7.5% overall had been prescribed medication for emotional or behavioral difficulties during the preceding 6 months. By U.S. Census region, the percentages were 9.3% in the South, 8.0% in the Midwest, 6.3% in the Northeast, and 5.0% in the West.

**Source:** National Health Interview Survey, 2011–2012. Available at http://www.cdc.gov/nchs/nhis.htm.

**Reported by:** LaJeana D. Howie, MPH, lhowie@cdc.gov, 301-458-4611; Patricia N. Pastor, PhD; Susan L. Lukacs, DO.

